# Notes and News

**Published:** 1900-06-30

**Authors:** 


### Notes and News.

The late Mrs. MacDonald, of Kepplestone, has bequeathed £1,000 to the Aberdeen Royal Infirmary, the authorities of which have endowed an In Memoriam bed with the legacy. It will be called the Hope MacDonald bed. Mrs. MacDonald was a generous donor to the infirmary during her lifetime.

The Prince of Wales has laid the foundation-stone of the new Royal Infirmary, Newcastle. The new institution will be very superior to the old one, both in outward appearance and all arrangements. There will be eight ward pavilions, administration block, operating theatre, mortuary, chapel, and nurses' home. The situation is an admirable one.

The Select Committee of the House of Commons on the Exemption of Hospitals from Rates has commenced its inquiries. The legal adviser to the Local Government Board, Mr. Adrian, has put before the Committee the previous history of attempts at amendment, together with the present position of the law on the subject. It appears that by a local Act the London Hospital is exempt from rates.

Italy is fully awakened to the need of strenuous measures to combat tuberculosis. The Church is now co-operating. Special attention is to be devoted to the cleansing and disinfecting of the churches, a measure more especially necessary in Roman Catholic countries, where they are resorted to daily by numbers of the poorer classes, who deposit not only merchandise, but undesirable burdens, such as soiled linen, on the flooring whilst occupied in their devotions. We learn that the heads of the Church are to meet to discuss what active part they can take in the crusade against consumption.

A curious incident is reported from Chicago. A free dispensary was attacked on May 31st by an infuriated crowd of Bohemians and Lithuanians, who had got the idea into their heads that human bodies were dissected in the place. A boy had been lost, and someone set the report going that he had been waylaid and killed by the doctors, when straightway a mob of several hundred people set to work to demolish the building. Nice place Chicago! But we have heard of English mobs doing things quite as crazy in the wrecking of small-pox hospitals. Of course, the child was all right.

Mr. H. Beerbohm Tree distributed prizes to the students at Charing Cross Hospital Medical School on Tuesday last. Mr. Tree, in addressing the students, urged them to make a continual progress in medical science, and take advantage of all the aid their generation afforded them.

The clergy in the various metropolitan places of worship did their best by eloquent pleading to emphasise the wants of the hospitals. Archdeacon Sinclair preached at St. Paul's, the Dean of Westminster at the Abbey, Canon Ainger at the Temple Church, and the Bishop of Southwark at St. Saviour's, Southwark.

A number of invalided soldiers have been sent at very short notice to the Woodhall Spa Hospital. Earlier in the year an offer of accommodation was declined by the War Office. Nevertheless the authorities consented to receive patients in response to a request by telegram from the War Office last week. To do this it was necessary to equip an entire ward, which was done by utilising the hospital board-room, and all was complete in 48 hours. The cases sent are all bad ones, likely to benefit by the baths.

The next competition for the "Howard Medal" of the Royal Statistical Society will take place in the ensuing session. The essays must be sent in on or before June 30th, 1901. In addition to the medal, a grant of £20 will be awarded to the writer who may be the successful competitor. The subject is "The History and Statistics of Tropical Diseases, with Especial Reference to Bubonic Plague." Rules and explanations may be obtained at the office of the Society, 9, Adelphi Terrace, Strand, W.C.

The *Daily Mail* has decided that a hospital and convalescent camp is needed for the sick and wounded soldiers from South Africa. In connection with the Absent-minded Beggar Relief Corps the proprietors have determined to establish and maintain a hospital for 500 soldiers through this organisation. The work done by the *Daily Mail* has been very prompt and effective, and it is not to be wondered at that it expresses the opinion that it can execute a task very much quicker than the Government machinery. Lord Lansdowne has given his sanction to the scheme, the hospital will be placed under a military medical officer, and Princess Louise will be president. The site chosen is at Alton, in Hampshire.

An extraordinary charge against the medical staff of the Coventry and Warwickshire Hospital was reported as having been made at the recent general meeting of the hospital by the mayor. Happily the house surgeons were able to meet the complaint with an immediate and emphatic denial, which, owing to its publicity at a general meeting, should prevent the charge ever being circulated again. The Mayor said he had been informed that patients about to be operated upon were placed under chloroform before the arrival of the hon. medical officer at the hospital, and that in one instance a patient was placed three times under chloroform whilst thus waiting. The emphatic denial of the house surgeons, of course, satisfied the meeting; but the charge is so grave that we think the medical officers would have been justified had they demanded the name of their accuser, and a subsequent apology from him.

